# New somatic *TERT* promoter variants enhance the Telomerase activity in Glioblastoma

**DOI:** 10.1186/s40478-020-01022-4

**Published:** 2020-08-25

**Authors:** Tiziana Pierini, Carlotta Nardelli, Anair Graciela Lema Fernandez, Valentina Pierini, Fabrizia Pellanera, Valeria Nofrini, Paolo Gorello, Martina Moretti, Silvia Arniani, Giovanni Roti, Paolo Giovenali, Marco Lupattelli, Giulio Metro, Carmen Molica, Corrado Castrioto, Rodolfo Corinaldesi, Maria Elena Laurenti, Stefano Ascani, Cristina Mecucci, Roberta La Starza

**Affiliations:** 1 Molecular Medicine Laboratory, Centro di Ricerche Emato-Oncologiche (C.R.E.O.), S. Maria della Misericordia Hospital, University of Perugia, P.le Menghini 9, 06132 Perugia, Italy; 2 Hematology and Center of Bone Marrow Transplants, Medicine and Surgery Department, University and Hospital of Parma, Via Gramsci 14, 43126 Parma, Italy; 3 Diagnostic Cytology and Histology Unit, S. Maria della Misericordia Hospital, P.le Giorgio Menghini 8/9, 06132 Perugia, Italy; 4 Division of Radiotherapy, S. Maria della Misericordia Hospital, P.le Giorgio Menghini 8/9, 06132 Perugia, Italy; 5 Medical Oncology, S. Maria della Misericordia Hospital, P.le Giorgio Menghini 8/9, 06132 Perugia, Italy; 6 Division of Neurosurgery, S. Maria della Misericordia Hospital, P.le Giorgio Menghini 8/9, 06132 Perugia, Italy; 7 Pathology Unit, S. Maria Hospital, V. Tristano di Joannuccio, 05100 Terni, Italy

**Keywords:** *TERT*, Gliomas, Gain-of-function mutation, ETS and Krüppel transcription factors

## Abstract

The catalytic activity of human Telomerase Reverse Transcriptase (TERT) compensates for the loss of telomere length, eroded during each cell cycle, to ensure a correct division of stem and germinal cells. In human tumors, ectopic *TERT* reactivation, most frequently due to hotspot mutations in the promoter region (*TERT*p), i.e. c.1-124 C > T, c.1-146 C > T, confers a proliferative advantage to neoplastic cells. In gliomas, *TERT*p mutations (*TERT*p^mut^) mainly occur in oligodendroglioma and glioblastoma. We screened, for *TERT*p hotspot mutations, 301 adult patients with gliomas and identified heterozygous mutations in 239 cases: 94% of oligodendroglioma, 85% of glioblastoma, and 37.5% of diffuse/anaplastic astrocytoma. Besides the recurrent c.1-124 C > T and c.1-146 C > T, two cases of glioblastoma harbored novel somatic *TERT*p variants, which consisted of a tandem duplications of 22 nucleotides, i.e. a *TERT*p c.1-100_1-79dup and *TERT*p c.1-110_1-89, both located downstream c.1-124 C > T and c.1-146 C > T. In silico analysis predicted the formation of 119 and 108 new transcription factor’s recognition sites for *TERT*p c.1-100_1-79dup and *TERT*p c.1-110_1-89, respectively. *TERT*p duplications (*TERT*p^dup^) mainly affected the binding capacity of two transcription factors’ families, i.e. the members of the E-twenty-six and the Specificity Protein/Krüppel-Like Factor groups. In fact, these new *TERT*p^dup^ significantly enhanced the E-twenty-six transcription factors’ binding capacity, which is also typically increased by the two c.1-124 C > T/c.1-146 C > T hotspot *TERT*p^mut^. On the other hand, they were distinguished by enhanced affinity for the Krüppel proteins. The luciferase assay confirmed that *TERT*p^dup^ behaved as gain-of-function mutations causing a 2,3-2,5 fold increase of *TERT* transcription. The present study provides new insights into *TERT*p mutational spectrum occurring in central nervous system tumors, with the identification of new recurrent somatic gain-of-function mutations, occurring in 0.8% of glioblastoma *IDH*-wildtype.

**Electronic supplementary material:**

The online version of this article (10.1186/s40478-020-01022-4) contains supplementary material, which is available to authorized users.

## Introduction

The abnormal reactivation of human Telomerase Reverse Transcriptase (TERT) is a common hallmark of human solid tumors. Although it may be caused by several mechanisms, i.e. methylation, mutations, rearrangements/fusions, and DNA copy number amplifications, *TERT* promoter (*TERT*p) methylation, and gain-of-function mutations are the most frequent [2, 28]. In particular, two recurrent hotspot mutations are respectively located at -124 (*TERT*p^-124^) and -146 (*TERT*p^-146^) base pairs (bp), from the *TERT* ATG start site [2, 10–12, 28]. Both mutations, generated from a cytidine to thymidine dipyrimide transition (C > T), are usually heterozygous, mutually exclusive, and produce an identical 11 bp ‘CCCCTTCCGGG’ sequence, resulting in the creation of de novo consensus binding motifs for E-twenty-six (ETS) transcription family members. These new binding sites recruit a larger number of ETS factors, enhancing the transcription of *TERT* [3].

*TERT* promoter mutations (*TERT*p^mut^) typically occur in tumors that arise from low self-renewal tissue, such as melanomas, thyroid, hepatobiliary carcinoma, and central nervous system (CNS) tumors, with a variable frequency, that range from 15 to 90% of cases, in diverse histological subtypes [10, 14, 28]. In CNS tumors, *TERT*p^mut^ are typically associated with glioblastoma (GBM) (70–80%) and oligodendroglioma (ODG) (60–70%), whereas their frequency decreases in other glioma subtypes, such as diffuse/anaplastic astrocytoma (DA/AA) (30–40%), medulloblastoma (20-30%), and meningioma (about 7%) [10, 25, 27]. Although the clinical value of *TERT*p^mut^, in refining the diagnostic classification of gliomas, is widely accepted [6], its role as prognostic/predictive biomarker is still largely debated. *TERT*p^mut^ have been associated with a poor disease outcome in GBM *IDH*-wildtype (GBM *IDH*^wt^), but there is no full agreement on its impact on DA/AA [6, 15, 16, 22, 24, 29]. It is worth noting, however, that DA/AA *IDH*-wildtype (DA/AA *IDH*^wt^) harboring genomic abnormalities typically associated with GBM, i.e. *TERT*p mutations, or *EGFR* amplification, or gain of whole chromosome 7 in combination with monosomy of chromosome 10, have a clinical outcome similar to, or only slightly longer, than GBM [4]. Thus, the cIMPACT NOW (Update 3) recommended to use one of these molecular criteria to classify this subgroup of astrocytomas as “diffuse astrocytic glioma, *IDH*-wildtype, with molecular features of glioblastoma, WHO grade IV” and to revise the classification of DA/AA *IDH*^wt^, accordingly [4].

Herein, we report two new *TERT*p mutations that were identified in two patients with GBM *IDH*^wt^. Both these new variants originated from the duplication of a stretch of 22 nucleotides at *TERT*p (*TERT*p^dup^) and, although slightly different, shared an overlapping sequence of 12 nucleotides. We demonstrated the somatic nature of one of these *TERT*p^dup^ and that, enhancing the binding affinity for ETS transcription factors (TFs), they both elicit the *TERT* transcription, thus widening the spectrum of recurrent gain-of-function mutations of *TERT*p in GBM.

## Case presentation

### Cohort

The study was carried out on a cohort of 301 patients, affected by primary CNS tumours, and referred to our laboratory during the last 10 years (Table 1). There were 175 males and 126 females (ratio 1.4:1) with a median age of 64 (range age: 20-86). According to the WHO 2016, the diagnosis was: grade II DA *IDH*^wt^ (6 cases) and DA *IDH*-mutant (DA *IDH*^mut^) (10 cases); grade III AA *IDH*^wt^ (6 cases) and AA *IDH*^mut^ (= 10); grade IV GBM *IDH*^wt^ (= 241) and GBM *IDH*^mut^ (= 10); grade II/III ODG (= 15). Three patients had a diagnosis of uncommon glioma (Table 1). The study was approved by Institutional Bioethics Committee (University of Perugia and Santa Maria della Misericordia Hospital of Perugia-Italy, Protocol no.2843/16); all patients gave informed consent for sample collection and molecular analyses, in agreement with the Declaration of Helsinki.

**Table 1:** Epidemiological and clinical features of our cohort of patients

Epidemiological-clinical data		
Total cohort		301
*Gender*	Male	175 pts (58.1%)
	Female	126 pts (41.9%)
	M:F	1.4
*Age (years)*	Range	20-86
	Median	64
	< 30 years	10 pts (3.3%)
	≥ 30 years	291 pts (96.7%)
*Diagnosis (WHO 2016)*		
*Common Gliomas*	Diffuse astrocytoma, *IDH*-wt (grade II)	6
	Diffuse astrocytoma, *IDH*-mut (grade II)	10
	Anaplastic astrocytoma, *IDH*-wt (grade III)	6
	Anaplastic astrocytoma, *IDH*-mut (grade III)	10
	Glioblastoma, *IDH*-wt (grade IV)	241
	Glioblastoma, *IDH*-mut (grade IV)	10
	Oligodendroglioma, *IDH*-mut and 1p/19q-codeleted (grade II)	7
	Anaplastic oligodendroglioma, *IDH*-mut and 1p/19q-codeleted (grade III)	8
*Uncommon Gliomas*	Pilocytic astrocytoma (grade I)	1
	Pleomorphic xanthoastrocytoma (grade II)	1
	Anaplastic pleomorphic xanthoastrocytoma (grade III)	1
*Anatomic location*	Frontal	97
	Frontal-parietal	15
	Frontal-temporal	5
	Parietal	39
	Parietal-occipital	10
	Temporal	84
	Temporal-parietal	17
	Temporal-occipital	3
	Occipital	9
	Cerebellar hemisphere	4
	Corpus callosum	2
	Thalamus	1
	Pituitary gland	1
	Insular	1
	Multicentric	13

pts, patients; wt, wildtype; mut, mutant

### Index cases

**Fig. 1:**
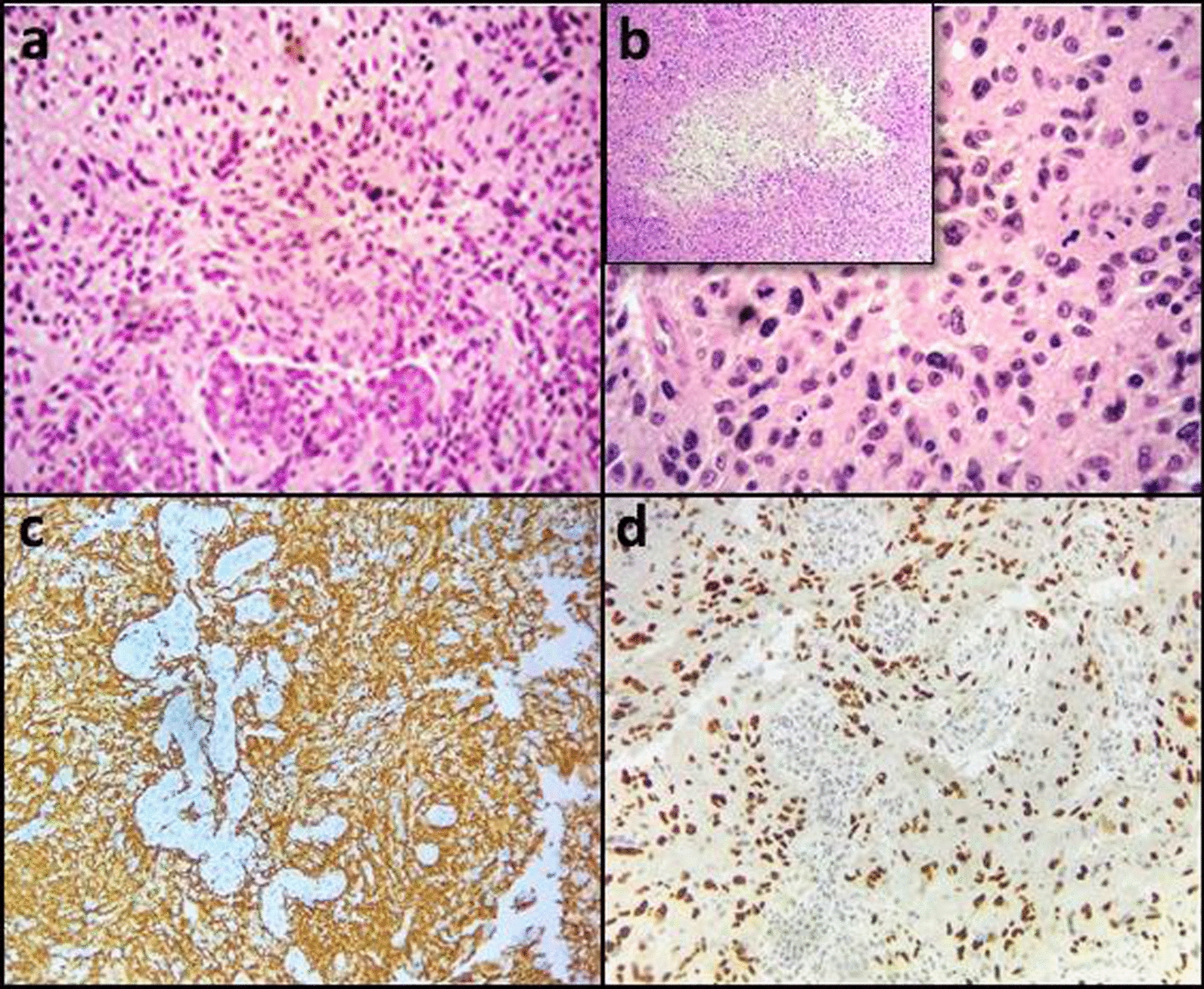
Histological and immunohistochemical analysis in patient UPN#171 **a** Hematoxylin/Eosin staining (original magnification 200X): enlarged neoplastic cells with multiple, often bizarre, hyperchromatic nuclei and high number of mitoses. Vascular proliferation, as seen in these “glomeruloids” (lower half of the image), is a specific pattern of microvascular growth; **b** Hematoxylin/Eosin staining (original magnification 400X): multiple mitotic figures are evident in the middle field. “Geographic pattern” of necrosis (detail in insert panel b); **c** Positive GFAP staining highlights high neoplastic cells with astrocytic differentiation; **d** Intense and diffuse nuclear TP53 staining

A 71-year-old male (UPN#131) had a left frontal lesion of 24 mm diameter, partially infiltrating the corpus callosum; the second case (UPN#171), a male of 78 years, presented with a right frontal lesion. Histopathology and immunohistochemistry were consistent with a diagnosis of GBM *IDH*^wt^, in both patients. In case UPN#131, neoplastic cells showed marked cytoplasmic and nuclear pleomorphism; there was a discrete number of atypical mitotic figures, widespread necrosis, a diffuse GFAP positivity (100%), and few neoplastic elements (20%) with strong nuclear TP53 stain. Case UPN#171, was characterized by striking atypia of neoplastic cells, diffuse necrosis, vascular proliferation, strong and diffuse positivity for GFAP and nuclear TP53 (> 70%) (Fig. 1). No *IDH1*/*IDH2* hotspot mutations were detected, while both cases showed *MGMT* promoter methylation. Monosomy of chromosome 10 co-occurred with *EGFR* amplification (UPN#131) or with gain of the whole chromosome 7 (UPN#171).

## Materials and methods

### *TERT* promoter mutational analysis

Genomic DNA was extracted from Formalin-Fixed Paraffin-Embedded (FFPE) tumor tissue and from peripheral blood (PB) by QIAamp DNA FFPE and AllPrep DNA/RNA kits, respectively, following the manufacturer’s instructions (QIAGEN, Milan, Italy). Hotspot *TERT*p^mut^ were investigated by Sanger sequencing using ABI 3500 Genetic analyzer instrument (Applied Biosystems, Monza, Italy). Primers were reported in Table S1 (Additional file 1: Table S1) and referred to GRCh37 genomic coordinate system (NM_000005.9, for regulatory core promoter 274 bp) (www.ncbi.nlm.nih.gov/gene [20], www.ensembl.org/Homo_sapiens [7]). Sequences’ alignments and their analyses were supported by Clustal Omega (www.ebi.ac.uk/Tools/msa/clustalo), Ensembl (http://www.ensembl.org/Homo_sapiens) [7], and COSMIC (https://cancer.sanger.ac.uk/cosmic) websites [5].

### In silico *TERT*p^mut^ functional analysis: JASPAR tool

This bioinformatic tool estimates the binding affinity and the number of TFs binding sites for the input sequence provided in FASTA format. A relative threshold score of 80% and Δ relative score ≥ 0.05 (mutant’s relative score—wildtype’s relative score) were chosen to define the statistically significant changes induced by *TERT*p^mut^, as previously reported [1]. The JASPAR CORE predicted the effects of the four different *TERT*p^mut^ that we detected in our patients, i.e. the two new *TERT*p^dup^, the *TERT*p^-124^, and the *TERT*p^-146^, on TFs binding capacity (JASPAR CORE Collection 2020; http://jaspar.genereg.net, 8th version [8, 13]). JASPAR was also used to analyze two *TERT*p^dup^, which have been previously reported in a case of MDS (c.1-110_1-101dup) and in a case of thyroid cancer (c.1-104_1-83dup) [21, 23]. According to JASPAR data, we used the Venn diagram to plot TFs for which a significant enhanced probability of binding capacity, or an increase of the number of binding sites, was predicted (http://bioinformatics.psb.ugent.be/webtools/Venn/).

### In vitro *TERT*p^mut^ functional study: luciferase assay

To study the effect of *TERT*p^mut^ on the expression of *TERT*, a luciferase assay was done for the *TERT*p^dup^ detected in case UPN#171, the *TERT*p^-146^ (UPN#205), and the *TERT*p^-124^ (UPN#216). The *TERT*^dup^ of case UPN#131 could not be studied due to lack of material. A *TERT*p wildtype (*TERT*p^wt^) construct, already available in the laboratory, was also used as reference (Additional file 2: Table S2) [21]. *TERT* core promoter (310 bp) was amplified with specific primers reported in Table S3 (Additional file 3: Table S3), introducing cleavage sites for BglII (forward) and HindIII (reverse) restriction enzymes. Then, *TERT*p^mut^ constructs were inserted in pGEM-T easy plasmid (Promega, Madison WI, USA) and cloned in Electromax DH10BT1 cells (Invitrogen, Milan, Italy) to increase the amount of mutant DNA. Finally, the inserts were subcloned in pGL4.10[luc2] vectors (Promega, Madison WI, USA) upstream of *LUC2* gene, encoding for luciferase enzyme of Photinus Pyralis and resequenced. An empty pGL4.10[luc2] vector was also used as negative control. Luciferase assay was performed using the GBM U87-MG cell line, maintained in Dulbecco’s Modified Eagle Medium (Thermo Fisher Scientific, Monza, Italy) with 10% fetal bovine serum, and 0.5% streptomycin/penicillin at 37 °C/5% CO_2_. U87-MG cells were seeded in a 6-multiwell plate (3 × 10^5^ cells/ml), co-trasfected with 3 µg of modified pGL4.10[luc2] plasmids and with 1:10 of pGL4.74[hRluc/TK], a vector containing the luciferase gene of Renilla Reniformis, by Viafect Transfection Reagent (Promega Madison WI, USA). After 24-h incubation, cells were lysed and fluorescence emission was assessed using Dual-Glo Luciferase assay kit (Promega) following manufacturer’s instructions. All experiments were performed in triplicate, in three independent experiments.

## Results

### New somatic *TERT* promoter variants

*TERT*p^mut^ were detected in 239/301 cases (79.4%), including 14/15 ODG (93%), 12/32 DA/AA (37.5%), and 213/251 GBM (84.8%) (Additional file 4: Table S4). In GBM (= 213) and DA/AA (= 12), *TERT*p^mut^ were prevalent in *IDH*^wt^ cases (209/241 GBM *IDH*^wt^ vs 4/10 GBM *IDH*^mut^; 10/12 DA/AA *IDH*^wt^ vs 2/20 DA/AA *IDH*^mut^) (Chi square, P < 0.001) (Additional file 5: Table S5). Thus, in agreement with the diagnostic criteria recommended by the cIMPACT-NOW (Update 3), the 10 DA/AA *IDH*^wt^ with *TERT*p^mut^ were referred to as “diffuse astrocytic glioma, *IDH*^-^wildtype, with molecular features of glioblastoma, WHO grade IV” [4].

In GBM *TERT*p^mut^ there was a significant enrichment of cases harbouring *EGFR* amplification (46% vs 17%) (Chi square, *P* = 0.001) and/or monosomy 10/*PTEN* deletions (84% vs 37.5%) (Chi square, P < 0.0001). Likewise, *EGFR* amplification or gain of whole chromosome 7 in combination with monosomy 10, occurred in 6/10 (60%) of *TERT*p^mut^ DA/AA *IDH*^wt^.

**Fig. 2:**
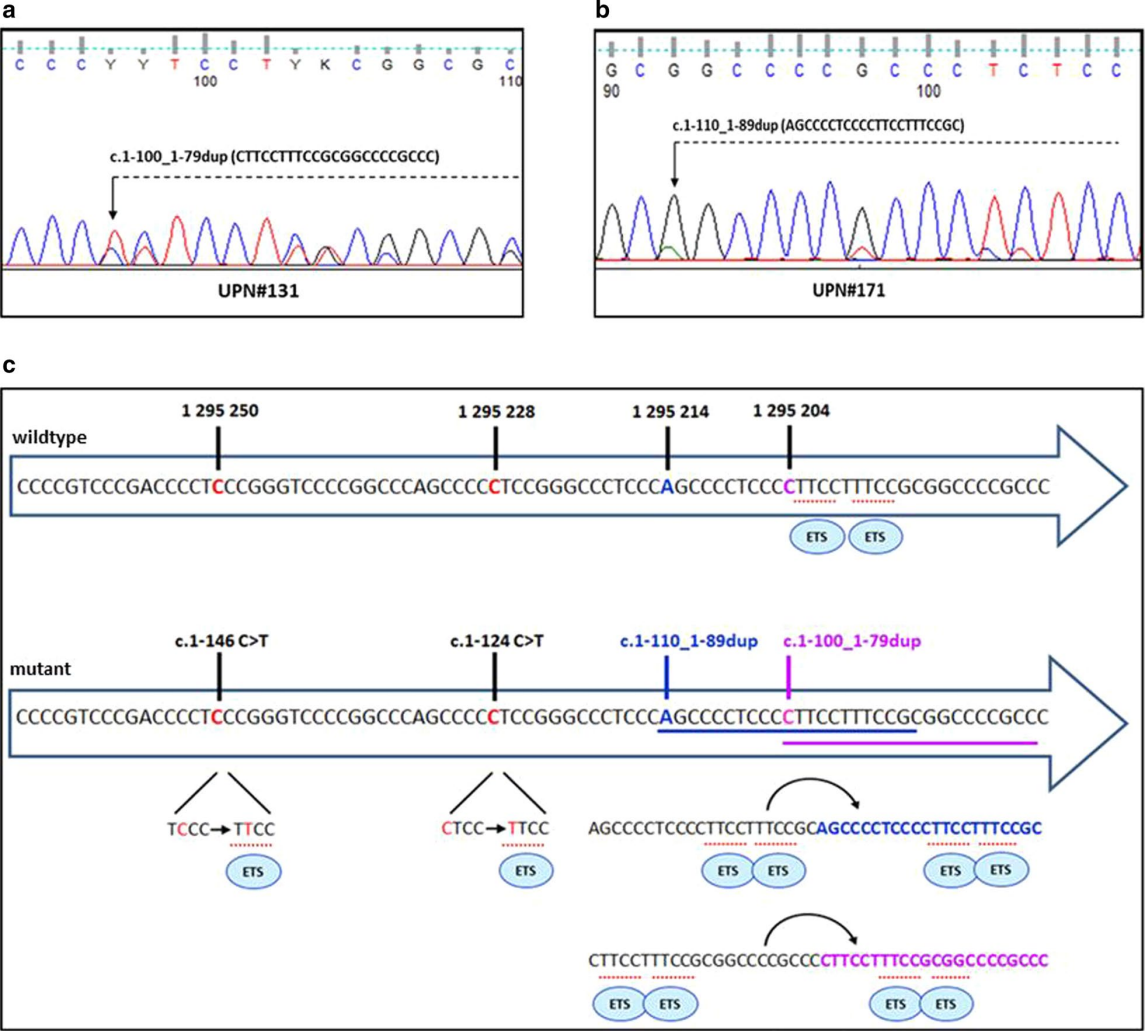
Schematic representation of *TERT*p mutations: **a** *TERT* promoter electropherogram in case UPN#131. The arrow indicates the start point of the c.1-100_1-79dup; **b** *TERT* promoter electropherogram in case UPN#171. The arrow indicates the start point of the c.1-110_1-89dup; **c** Overview of all *TERT*p variants detected in our cases. Upper arrow: wildtype *TERT* core promoter with the normal location of ETS binding sites. The vertical black lines indicate the genomic positions of *TERT*p variants. Lower arrow: positions and types of *TERT*p variants and their predicted effects on transcription factors binding sites

The most common variant, *TERT*p^-124^ was detected in 172 cases while the *TERT*p^-146^ was found in 65 cases. *TERT*p^mut^ were mutually exclusive, heterozygous, and equally distributed among the different histological subtypes (Additional file 5: Table S5). Besides the *TERT*p^-124^ and *TERT*p^-146^, we uncovered two new *TERT*p variants in two cases of GBM *IDH*^wt^ (UPN#131 and UPN#171). These novel *TERT*p^mut^ consisted of a 22 nucleotide tandem duplication, occurring in a genomic region starting at 100 and 110 bp, from the ATG starting site, i.e. c.1-100_1-79dup (*TERT*p^-100-79^), in case UPN#131, and c.1-110_1-89dup (*TERT*p^-110-89^), in case UPN#171 (Fig. 2a, b) (www.ncbi.nlm.nih.gov/gene, www.ensembl.org/Homo_sapiens, cancer.sanger.ac.uk/cosmic) [5, 7, 20]. They shared a region of duplication of 12 nucleotides, from 1–100 to 1–89 nucleotides from the ATG start site. The absence of *TERT*p^-100-79^ in the PB DNA, demonstrated the somatic origin of this variant in case UPN#131.

### In silico analysis predicts *TERT*p^mut^ effects

In silico analysis predicted that both *TERT*p^dup^ created new binding sites, i.e. 119 for *TERT*p^-100-79^ and 108 for *TERT*p^-110-89^, which were respectively recognized by 65 and 53 TFs. Instead, *TERT*p^-124^ and *TERT*p^-146^ were predicted to increase the binding affinity for 40 and 43 sites, and to enhance the probability of binding for 28 and 29 TFs, respectively (Additional file 6: Table S6). Although all *TERT*p^mut^ affected the binding sites for diverse families of TFs, the ETS group emerged as one of the most frequently involved: 18/65 (28%) in *TERT*p^-100-79^, 18/53 (34%) for *TERT*p^-110-89^, 23/28 (82%) in *TERT*p^-124^, and 25/29 (86%) in *TERT*p^-146^, (Fig. 2c, Additional file 7: Table S7). Other recurrently involved TFs in *TERT*p^dup^ variants were the Specificity Protein/Krüppel-Like Factor (Sp/KLF) family, i.e. 19/65 (29%) in *TERT*p^-100-79^ and 16/53 (30%) in *TERT*p^-110-89^, and the More than 3 adjacent zinc finger factors family (12/65 in *TERT*p^-100-79^ and 7/53 *TERT*p^-110-89^) (Additional file 7: Table S7).

**Fig. 3:**
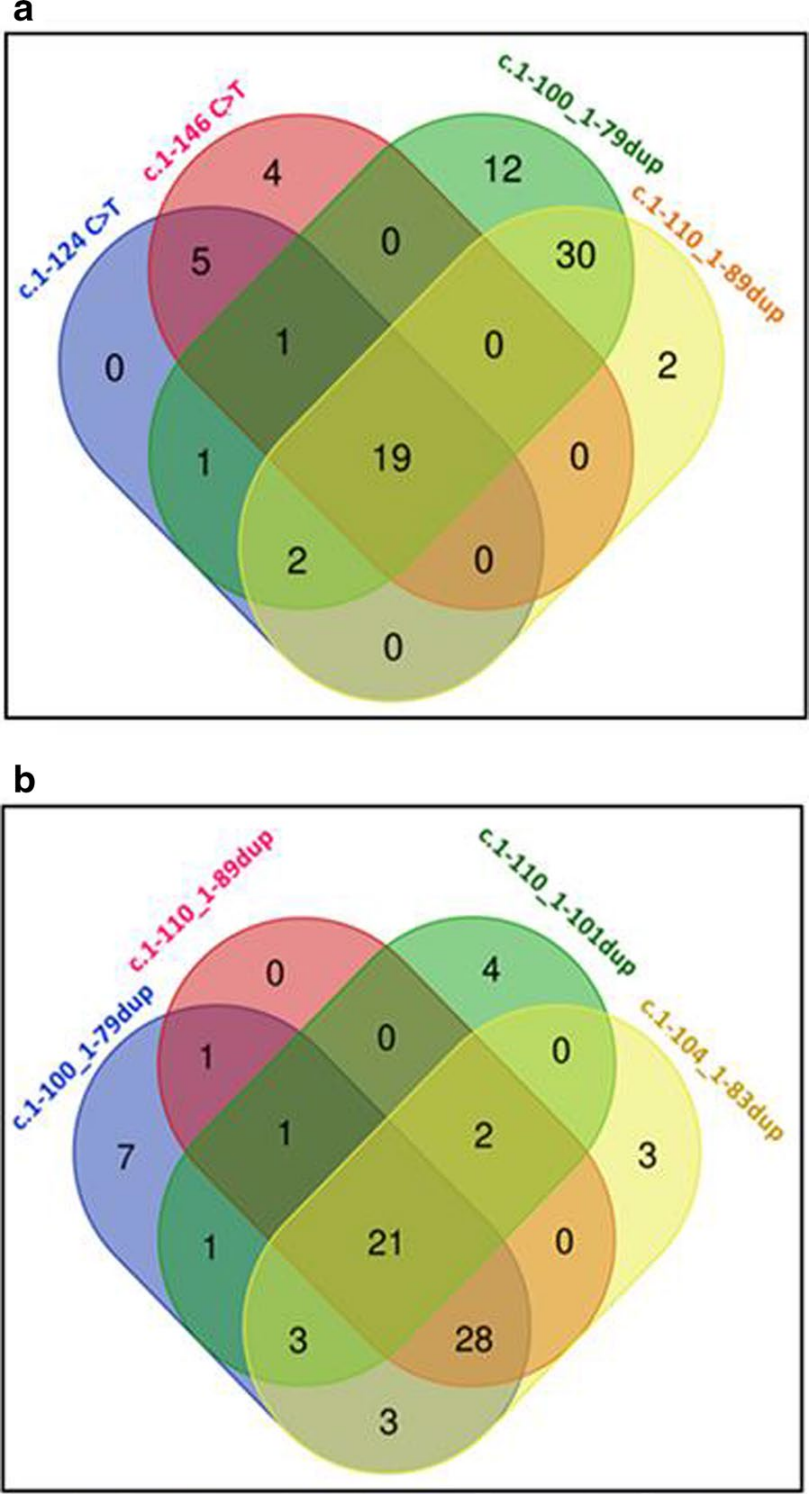
The Venn diagrams show all possible relations among: **a** four *TERT*p variants reported in our cases (refer to Additional file 8: Table S8) and; **b** *TERT*p^dup^ described in this study (c.1-100_1-79dup and c.1-110_1-89dup) and those reported in literature (c.1-104_1-83dup and c.1-110_1-101dup) (refer to Additional file 11: Table S11)

The Venn diagram showed a close inter-relationship between all *TERT*p mutations. Namely, all *TERT*p mutations shared an increase of the binding affinity, or the number of binding motifs, for 19 common TFs (Fig. 3a), including 18 ETS members (ETS1, ETS2, ERG, ELK1, ETV6, FLI1, ELK4, SPIB, ELF1, ELF3, ETV4, ETV1, FEV, EHF, ETV5, ELF5, SPI1, and GABPA) and TEAD1 (Fig. 3a; Additional file 8: Table S8). The Venn diagram also showed that the new *TERT*p^dup^ were characterized by the exclusive involvement of 30 common TFs. Specifically, there were 16 Sp/KLF members, i.e. KLF2, KLF3, KLF4, KLF5, KLF10, KLF11, KLF14, KLF15, KLF16, SP1, SP2, SP3, SP4, SP8, SP9, and EGR1, (Fig. 3a, Additional file 8: Table S8) and 14 TFs that belong to 9 different families (Fig. 3a, Additional files 7 and 8: Tables S7 and S8). Matching our *TERT*p^dup^ with the two cases of *TERT*p^dup^ previously reported (Additional files 9 and 10: Tables S9 and S10) [21, 23], JASPAR predicted that all variants determined an increase of binding sites for 21 common TFs, and confirmed that the Sp/KLF family was the most frequently involved (14/21) (Fig. 3b, Additional file 11: Table S11).

### In vitro analysis confirms the increasing of *TERT* transcriptional activity induced by its promoter mutations

**Fig. 4:**
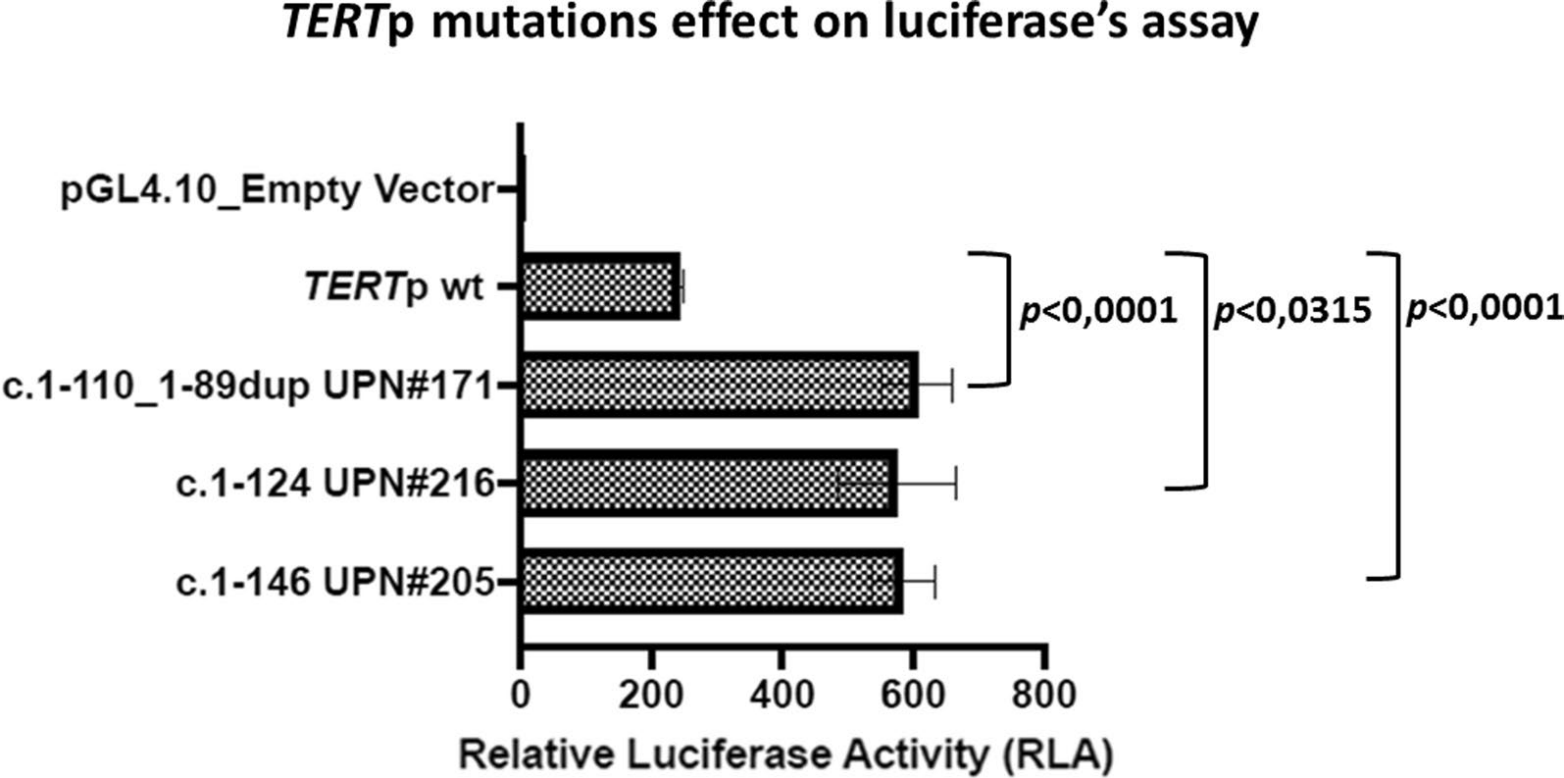
Luciferase assay. The histogram reports the relative luciferase activities (RLA) of *TERT*p wildtype and for the variants c.1-110_1-89dup, c.1-124 C > T, and c.1-146 C > T. *p* value refers to probability obtained using Mann–Whitney U test

In vitro luciferase assay was carried out to evaluate whether the new *TERT*p^-110-89^ variant induced an increase of *TERT* transcriptional activity, enhancing its expression, similarly to *TERT*p^-124^ and *TERT*p^-146^ [12, 21]. In Table S12 (Additional file 12: Table S12) we reported raw data referred to the fluorescence emission values, expressed in Relative Luciferase Activity (RLA), of both Photinus Pyralis and Renilla Reniformis luciferase enzymes, for all samples. Our experiments demonstrated that all three variants caused a significant increase of *TERT* transcription by 2.3-2.5 fold than wildtype (*TERT*p^-110-89^ vs *TERT*p^wt^: *P *< 0,0001; *TERT*p^-124^ vs *TERT*p^wt^: *P *< 0,0315; *TERT*p^-146^ vs *TERT*p^wt^: *P *< 0,0001; Mann–Whitney U test) (Fig. 4). On the other hand, no differences on the levels of *TERT* expression were present between the diverse *TERT*p variants, indicating they may all behave as gain-of-function mutations, likely exerting the same consequences on *TERT* transcription.

## Discussion

Abnormal genomic events that alter telomere elongation are common in gliomas. Particularly, mutually exclusive mutations affect the *TERT* or the *ATRX* chromatin remodeler (*ATRX*) genes, a critical regulator of telomere homeostasis by chromatin remodeling [9].

Our studies, on a cohort of 301 patients, confirmed previous data on the incidence and distribution of *TERT*p^mut^ in diverse subtypes of CNS tumors. As expected, we found that *TERT*p^mut^ were highly recurrent in ODG and GBM, and less frequent in DA/AA (Additional file 4: Table S4). *TERT*p^mut^ were significantly enriched in GBM *IDH*^wt^ cases (83%) (Chi square, P < 0.001) (Additional file 5^5^: Table S5), where they mainly occurred together with *EGFR* amplification (Chi square, P = 0.001) and/or monosomy 10/*PTEN* deletions (Chi square, P < 0.0001). Similarly, in DA/AA, *TERT*p^mut^ were highly recurrent in *IDH*^wt^ cases, thus allowing the reclassification of 83% of these subgroup of astrocytomas as “diffuse astrocytic glioma, *IDH*-wildtype, with molecular features of glioblastoma, WHO grade IV” [4].

Besides the two known *TERT*p^-124^ and *TERT*p^-146^ variants, we uncovered two new *TERT*p variants in two cases of GBM *IDH*^wt^ (UPN#131 and UPN#171). These novel *TERT*p^mut^ consisted of a 22 nucleotide tandem duplication, sharing a duplicated region of 12 nucleotides, from 1–100 to 1–89, from the ATG start site. Hitherto, somatic *TERT*p^dup^ has been reported in three human tumors. The first one, a duplication of 41 nucleotides in the *TERT* core promoter, was detected in a case of ODG [3]. Afterwards, *TERT*p^dup^ were found in a case of myelodysplastic syndrome (MDS) (c.1-110_1-101dup) and in a case of papillary thyroid carcinoma (c.1-104_1-83dup) [21, 23]. Published *TERT*p^dup^ as well as our cases, are located in the same core promoter region, that span 1-110/1-79 bp from the ATG start site. Furthermore, they are all located downstream *TERT*p^-124^ and *TERT*p^-146^, i.e. at 13–23 nucleotides from *TERT*p^-124^ and 35-45 nucleotides from *TERT*p^-146^, in a region that contains the binding sites for the TFs modulating *TERT* transcription. Interestingly, in silico analysis predicted these new *TERT*^dup^ affect the transcriptional regulation of the gene through the creation of new binding sites for TFs that mainly belong to the ETS family (Fig. 2c, Additional file 7: Table S7). Likewise, an increased number of binding sites or an enhanced affinity for the ETS TFs, has been previously reported in a thyroid cancer harbouring a *TERT*p c.1-104_1-83dup variant, and in cases bearing *TERT*p^-124^ or *TERT*p^-146^ mutations [3, 10, 23]. Bioinformatic analyses were consistent with the luciferase data showing a significant increase of *TERT* expression in cells transfected with the new *TERT*p^-110-89^ variant as well as with the two recurrent *TERT*p^mut^.

Then, we sought to assess the possible inter-relationship between the four diverse *TERT*p mutations using the Venn diagram (Fig. 3a). All four *TERT*p variants were predicted to share an increase binding capacity for 18 ETS members (Fig. 3a; Additional file 8: Table S8), which included GABPA, a putative oncogene in GBM. Namely, in vitro studies on GBM cell lines have demonstrated that this transcription factor is needful in mediating the transcriptional reactivation of *TERT* dependent from *TERT*p^-124^ or *TERT*p^-146^ [3, 10, 19]. Besides ETS TFs, all *TERT*p variants affected the binding capacity for TEAD1, a protein that belongs to TEF-1-related factors family, and that has been demonstrated to act as a putative oncogene in GBM, favoring cell infiltration in vitro/in vivo models [26].

Although *TERT*p^-124^ and *TERT*p^-146^, and the new *TERT*p^-100-79^ and *TERT*p^-110-89^ variants, shared the same effects on the binding capacity for ETS members, the latters were characterized by the exclusive involvement of 30 TFs, mainly belonging to Sp/KLF family (Fig. 3a, Additional files 7 and 8: Tables S7 and S8). Sp/KLF TFs are involved in a plethora of cellular processes ranging from proliferation and differentiation, pluripotency and apoptosis, in normal and tumoral tissues [17].

Altogether these data support the hypothesis that the recruitment of ETS family TFs plays a pivotal role in mediating the reactivation of *TERT* transcription in human tumors bearing different types of *TERT*p^mut^. However, they also indicate that slight differences mark *TERT*p^dup^ variants, whose activities appear to be also dependent from Krüppel-related factors. Indeed, among the 21 TFs shared by all *TERT*p^dup^ (Fig. 3b), 14 belonged to Sp/KLF family (67%) as reported in Tables S10 and S11 (Additional files: 10 and 11). Hence, the precise definition of mutation-specific profiles would strengthen the definition of *TERT*-dependent oncogenesis mechanisms.

Our study contributes to enrich the spectrum of recurrent somatic *TERT*p^dup^ variants reporting, for the first time, two new gain-of-function mutations, i.e. *TERT*p^-100-79^ and *TERT*p^-110-89^, in 0.8% of GBM *IDH*^wt^ cases. These new mutations can be reliably detected by diagnostic assays used to investigate hotspot *TERT*p^-124^ and *TERT*p^-146^. Although the assessment of *TERT*p mutational status is not an essential diagnostic criterion, it can be a relevant information to assist histological diagnosis [18]. As a matter of fact, the status of *TERT*p, together with *IDH* mutations and 1p/19q co-deletion, classify gliomas in 5 distinct subcategories, i.e. triple negative, triple positive, cases with *IDH*/*TERT* mutations, and cases with a unique mutation (either *IDH* or *TERT)*, that are typified by unique demographic, clinical and biological characteristics [6]. Moreover, *TERT*p^mut^ has been proposed as one of the most relevant molecular marker to stratify DA/AA *IDH*^wt^ [4]. Thus, we consider that molecular testing of *TERT*p mutations should be included in the clinical work-up of GBM and DA/AA in order to provide a precise diagnosis: prospective multicentric studies, on large cohort of patients, will clarify the value of *TERT*p mutations as prognostic marker.

## Supplementary information

**Additional file 1: Table S1.** Primer set used for Sanger sequencing.

**Additional file 2: Table S2.**Samples used for in vitro luciferase assay.

**Additional file 3: Table S3.** Primer set used to create constructs for luciferase assay.

**Additional file 4: Table S4.** Incidence and distribution of *TERT*p variants in the main glioma subgroups.

**Additional file 5: Table S5.** Incidence and distribution of *TERT*p variants in glioma subtypes (according to WHO 2016 guidelines).

**Additional file 6: Table S6.** JASPAR analysis for the* TERT*p c.1-124 C>T, c.1-146 C>T and the new* TERT*p^dup^(c.1-100_1-79dup; c.1-110_1-89dup).

**Additional file 7: Table S7.** Transcription Factors predicted to be involved in* TERT*p variants.

**Additional file 8: Table S8.** Transcription Factors predicted to be involved in different* TERT*p variants.

**Additional file 9: Table S9.** JASPAR analysis for the two published* TERT*p duplications c.1-110_1-101dup and c.1-104_1-83dup [ref. 21, 23].

**Additional file 10: Table S10.** Transcription factors predicted to be involved in the * TERT*p^dup^ c.1-110_1-101dup and c.1-104_1-83dup [ref. 21, 23].

**Additional file 11: Table S11.** Transcription factors predicted to be involved in all* TERT*p duplications.

**Additional file 12: Table S12.** Luciferase assay: raw data.

## Data Availability

All data generated or analyzed during this study are included in this published article [and in its supplementary information files].

## Abbreviations

*TERT*: Telomerase Reverse Transcriptase
*TERT*p: *TERT* promoter
*TERT*p^mut^: *TERT* promoter mutation
*TERT*p^dup^: *TERT* promoter duplication
*TERT*p^-124^: c.1-124 *TERT* promoter mutation
*TERT*p^-146^: c.1-146 *TERT* promoter mutation
bp: base pair
ETS: E-twenty-six transcription factor
CNS: central nervous system
GBM: glioblastoma
ODG: oligodendroglioma
DA: diffuse astrocytoma
AA: anaplastic astrocytoma
GBM *IDH*^wt^: glioblastoma *IDH*-wildtype
DA *IDH*^wt^: diffuse astrocytoma *IDH*-wildtype
AA *IDH*^wt^: anaplastic astrocytoma *IDH*-wildtype
TFs: transcription factors
DA *IDH*^mut^: diffuse astrocytoma *IDH*-mutant
AA *IDH*^mut^: anaplastic astrocytoma *IDH*-mutant
GBM *IDH*^mut^: glioblastoma *IDH*-mutant
FFPE: formalin-fixed paraffin-embedded
PB: peripheral blood
MDS: myelodysplastic syndrome
*TERT*p^wt^: *TERT*p wildtype
*TERT*p^-100-79^: c.1-100_1-79dup
*TERT*p^-110-89^: c.1-110_1-89dup
Sp/KLF: Specificity Protein/Krüppel-Like Factor
RLA: relative luciferase activity
*ATRX*: *ATRX* chromatin remodeler
